# Supplementary material for: Content-rich biological network constructed by mining PubMed abstracts
Source: BMC Bioinformatics. 2004 Oct 8;5:147. doi: 10.1186/1471-2105-5-147 (PMC528731; doi:10.1186/1471-2105-5-147)
Supplement: Additional File 2 — The original results of the above study (non-essential files are deleted to keep the file size under the limit set by BMC bioinformatics). [file 1471-2105-5-147-S2.bz2 › chilibotAdditionalFile2/dip30/382ID8621424E9795/html/NTF3_NTRK2.html]

 


 **NTF3** and **NTRK2** 
  
Found 413 abstracts in PubMed, retrieved 30.  
 

 What does Google say? 
 PDF only 
| .edu only 

---

**Interactive relationship** (e.g. stimulation, inhibition, etc)

**Neutral relationship**- BDNF stimulated much stronger interactions of PLCgamma with Trk than NGF and NT 3  [ **NTF3** ]  in PC12 cells stably expressing TrkB  [ **NTRK2** ]  and cultured cerebral cortical neurons, respectively, although BDNF, NGF and NT 3  [ **NTF3** ]  induced similar levels of tyrosine phosphorylation of Trk.  Ref: 12419535 Brain Res, 2002
- Because TrkC like IR in bud cells is absent, and sparse in fibers intragemmally and perigemmally, NT 3  [ **NTF3** ]  may also bind to bud cell TrkB  [ **NTRK2** ]  so as to sustain fungiform gemmal cell viability post denervation.  Ref: 12454994 J Comp Neurol, 2003
- Here we show that p62 interacts not only with TrkA, but also with TrkB  [ **NTRK2** ]  and TrkC, which are the primary receptors for brain derived neurotrophic factor and neurotrophin 3  [ **NTF3** ] .  Ref: 12471037 J Biol Chem< MedlineTA>J Biol Chem, 2003
- In the gustatory system, NT3  [ **NTF3** ]  is unable to replace the actions of BDNF possibly because of a temporally selective expression of TrkB  [ **NTRK2** ]  in taste neurons.  Ref: 12620975 Development< MedlineTA>Development, 2003
- Nerve growth factor, brain derived neurotrophic factor and neurotrophin 3  [ **NTF3** ] , members of the neurotrophin family, bind to and activate TrkA, TrkB  [ **NTRK2** ]  and TrkC, respectively, members of the Trk receptor tyrosine kinase family, to exert various effects including promotion of differentiation and survival, and regulation of synaptic plasticity in neuronal cells.  Ref: 12419535 Brain Res, 2002

**Non-interactive relationship** (e.g. studied together, co-existance, homology, etc.)

- We also examined whether a non Trk B  [ **NTRK2** ]  neurotrophin ligand, neurotrophin 3  [ **NTF3** ]  NT 3  [ **NTF3** ] , is present within the follicle and has a possible role in oocyte maturation.  Ref: 12364436 J Clin Endocrinol Metab, 2002
- We found that 1 trk C, a receptor for neurotrophin 3  [ **NTF3** ]  NT 3  [ **NTF3** ] , is dominantly expressed in RCEC, but trk B  [ **NTRK2** ] , a receptor for brain derived neurotrophic factor, is dominantly expressed in RAEC.  Ref: 12745089 Biochem Biophys Res Commun, 2003
- In contrast, the expression levels of BDNF, NT 3  [ **NTF3** ] , trkB  [ **NTRK2** ]  and trkC were significantly higher at the rostral and caudal sites immediately adjacent to the maximal compression site.  Ref: 12774239 Acta Neuropathol Berl , 2003
- Cultured cortical progenitors express BDNF and NT 3  [ **NTF3** ] , as well as their receptors TrkB tyrosine kinase  [ **NTRK2** ]  receptor B and TrkC.  Ref: 12832539 J Neurosci, 2003
- Consistent with a trkB  [ **NTRK2** ] .FL mediated mechanism, the i.t. administration of another trkB  [ **NTRK2** ]  ligand, neurotrophin 4 5, also produces hyperalgesia while the trkC agonist neurotrophin 3  [ **NTF3** ] , which weakly cross reacts with trkB  [ **NTRK2** ] , has little effect.  Ref: 12435470 Pain, 2002
- The role of the neurotrophin ligands, brain derived neurotrophic factor and neurotrophin 3  [ **NTF3** ] , and their respective tyrosine kinase receptors, TrkB  [ **NTRK2** ]  and TrkC, in denervated taste buds is not known.  Ref: 12454994 J Comp Neurol, 2003
- In contrast, for anterior tongue fungiform bud cells, the degree of neurotrophin and receptor like IR was relatively less affected NT 3  [ **NTF3** ]  and TrkB  [ **NTRK2** ]  like IR were unchanged.  Ref: 12454994 J Comp Neurol, 2003
- Our results suggest that overexpression of BDNF, NT 3  [ **NTF3** ] , trkB  [ **NTRK2** ]  and trkC in motoneuron areas neighboring the site of mechanical compression may represent compensatory changes in response to the compromised neuronal function at the level of compression,.  Ref: 12774239 Acta Neuropathol Berl , 2003
- In single and double labeled immunofluorescent experiments, 09 taste bud sections from 13 hamsters were examined for immunopositive gemmal cells or nerve fibers using antibodies to BDNF and NT 3  [ **NTF3** ] , their respective receptors TrkB  [ **NTRK2** ]  and TrkC, and the neural marker ubiquitin c terminal hydrolase L 1 protein gene product 9.5.  Ref: 12454993 J Comp Neurol, 2003
- In each gemmal field, more than 75% of taste bud profiles showed immunopositivity to BDNF, NT 3  [ **NTF3** ] , and TrkB  [ **NTRK2** ] .  Ref: 12454993 J Comp Neurol, 2003
- The neurotrophins brain derived neurotrophic factor and neurotrophin 3  [ **NTF3** ] , as well as their respective tyrosine kinase receptors, TrkB  [ **NTRK2** ]  and TrkC, influence peripheral target cell innervation, survival, and proliferation.  Ref: 12454993 J Comp Neurol, 2003
- In this work the expression and anatomical localization of NGF, BDNF and NT 3  [ **NTF3** ]  and of corresponding high affinity receptors TrkA, TrkB  [ **NTRK2** ]  full length and truncated TR isoforms, TrkC and of the low affinity p75 receptor were assessed in surgical samples from adult human lung by reverse transcriptase polymerase chain reaction RT PCR, Western blot and immunohistochemistry.  Ref: 12791675 Am J Respir Cell Mol Biol< MedlineTA>Am J Respir Cell Mol Biol, 2003
- Increased neuron losses associated with the presence of truncated TrkB  [ **NTRK2** ]  were most severe in regions where neuron survival is most dependent on brain derived neurotrophic factor and neurotrophin 3  [ **NTF3** ] .  Ref: 12654337 Neuroscience, 2003
- In taste buds that survived on the posterior tongue, the degree of foliate bud cell BDNF, NT 3  [ **NTF3** ] , and TrkB  [ **NTRK2** ]  like IR, and circumvallate bud cell BDNF and NT 3  [ **NTF3** ]  like IR, significantly decreased compared with the unoperated side.  Ref: 12454994 J Comp Neurol, 2003
- Immunoreactivities for BDNF, NT 3  [ **NTF3** ] , trkB  [ **NTRK2** ]  and trkC were preferentially localized in the gray matter, particularly in the anterior horn cells.  Ref: 12774239 Acta Neuropathol Berl , 2003
- Western blot analysis confirmed these results, showing that the lacrimal gland expresses NGF, BDNF, NT3  [ **NTF3** ] , and NT4 as well as the NTrs TrkA, TrkB  [ **NTRK2** ] , and TrkC and the p75 protein.  Ref: 12882781 Invest Ophthalmol Vis Sci, 2003
- As the VMN undergoes sexual differentiation, we compared BDNF, NGF, NT 3  [ **NTF3** ] , and trkB  [ **NTRK2** ]  mRNA expression in the VMN in males and females at embryonic day 20 and PND 4, but found no differences between them.  Ref: 12824057 Brain Res Mol Brain Res, 2003
- To determine which of the neurotrophins NTs nerve growth factor NGF, brain derived neurotrophic factor BDNF, neurotrophin 3  [ **NTF3** ]  NT3  [ **NTF3** ] , and neurotrophin 4 5 NT4 and their receptors NTrs, TrkA, TrkB  [ **NTRK2** ] , TrkC, and p75, are present in the adult rat lacrimal gland.  Ref: 12882781 Invest Ophthalmol Vis Sci, 2003
- RT PCR showed positive bands at the appropriate sizes for NGF, BDNF, NT3  [ **NTF3** ] , and NT4, and for the receptors TrkA, TrkB  [ **NTRK2** ] , TrkC, and p75.  Ref: 12882781 Invest Ophthalmol Vis Sci, 2003
- In summary, our findings suggest that, unlike NT3  [ **NTF3** ]  and GDNF, TrkB  [ **NTRK2** ]  ligands are required and sufficient to promote the development of central noradrenergic neurons.  Ref: 12810600 Development, 2003
- However, despite overlapping expression of TrkB  [ **NTRK2** ]  and TrkC in many sensory ganglia, brain derived neurotrophic factor and neurotrophin 3  [ **NTF3** ]  null mutant mice display selective losses in neuronal subpopulations.  Ref: 12620975 Development< MedlineTA>Development, 2003
- New findings were RT PCR for neurotrophins and their receptors detected NT 3  [ **NTF3** ]  and NT 4 5 in alveolar macrophages, BDNF, NT 4 5, trkA, the truncated form of trkB  [ **NTRK2** ] , and trkC in lung homogenate, but no trks in alveolar macrophages, immunohistochemistry for neurotrophin receptors detected TrkA in capillary cells, the truncated form of TrkB  [ **NTRK2** ] , and TrkC in interstitial macrophages, immunoelectron microscopy for TrkC revealed expression of TrkC on the surface of interstitial macrophages, and in situ hybridization for neurotrophins detected BDNF in interstitial macrophages and alveolar type I cells, NT 3  [ **NTF3** ]  in alveolar macrophages, and NT 4 5 in alveolar and interstitial macrophages.  Ref: 12122447 Histochem Cell Biol, 2002
- For this purpose, serial immunohistochemical and immunoblot analyses were performed for the expression levels of endogenous brain derived neurotrophic factor BDNF, neurotrophin NT 3  [ **NTF3** ] , and their receptors, trkB  [ **NTRK2** ]  and trkC in 24 tip toe walking Yoshimura mice twy twy aged 12 24 weeks.  Ref: 12774239 Acta Neuropathol Berl , 2003
- Hence, NT 3  [ **NTF3** ] , BDNF, and TrkB  [ **NTRK2** ]  like IR in fungiform gemmal cells may reflect an autocrine capacity promoting survival.  Ref: 12454994 J Comp Neurol, 2003
- We have used immunofluorescence techniques with a panel of antibodies against known neurotrophin receptors trk A, trk B  [ **NTRK2** ] , trk C, p75NTR to map the locations of these receptors in the developing neuromuscular system of mice from our neurotrophin 3  [ **NTF3** ]  NT 3  [ **NTF3** ]  knockout colony.  Ref: 12141445 Int J Dev Biol, 2002
- Messenger RNAs encoding other neurotrophins, including nerve growth factor NGF and neurotrophin 3  [ **NTF3** ]  NT 3  [ **NTF3** ]  and the BDNF receptor trkB  [ **NTRK2** ] , were not selectively localized in neonatal VMN.  Ref: 12824057 Brain Res Mol Brain Res, 2003
